# Ticket to spawn: Combining economic and genetic data to evaluate the effect of climate and demographic structure on spawning distribution in Atlantic cod

**DOI:** 10.1111/gcb.14474

**Published:** 2018-10-30

**Authors:** Øystein Langangen, Leonie Färber, Leif C. Stige, Florian K. Diekert, Julia M. I. Barth, Michael Matschiner, Paul R. Berg, Bastiaan Star, Nils Chr. Stenseth, Sissel Jentoft, Joël M. Durant

**Affiliations:** ^1^ Centre for Ecological and Evolutionary Synthesis (CEES), Department of Biosciences University of Oslo Oslo Norway; ^2^ Department of Economics Heidelberg University Heidelberg Germany; ^3^ Zoological Institute University of Basel Basel Switzerland; ^4^ Centre for Coastal Research (CCR), Department of Natural Sciences University of Agder Kristiansand Norway

**Keywords:** climate change, demography, economic data, genetic data, size truncation, spawning distribution

## Abstract

Climate warming and harvesting affect the dynamics of species across the globe through a multitude of mechanisms, including distribution changes. In fish, migrations to and distribution on spawning grounds are likely influenced by both climate warming and harvesting. The Northeast Arctic (NEA) cod (*Gadus morhua*) performs seasonal migrations from its feeding grounds in the Barents Sea to spawning grounds along the Norwegian coast. The distribution of cod between the spawning grounds has historically changed at decadal scales, mainly due to variable use of the northern and southern margins of the spawning area. Based on historical landing records, two major hypotheses have been put forward to explain these changes: climate and harvesting. Climate could affect the distribution through, for example, spatial habitat shifts. Harvesting could affect the distribution through impacting the demographic structure. If demographic structure is important, theory predicts increasing spawner size with migration distance. Here, we evaluate these hypotheses with modern data from a period (2000–2016) of increasing temperature and recovering stock structure. We first analyze economic data from the Norwegian fisheries to investigate geographical differences in size of spawning fish among spawning grounds, as well as interannual differences in mean latitude of spawning in relation to changes in temperature and demographic parameters. Second, we analyze genetically determined fish sampled at the spawning grounds to unambiguously separate between migratory NEA cod and potentially smaller sized coastal cod of local origin. Our results indicate smaller spawners farther away from the feeding grounds, hence not supporting the hypothesis that harvesting is a main driver for the contemporary spawning ground distribution. We find a positive correlation between annual mean spawning latitude and temperature. In conclusion, based on contemporary data, there is more support for climate compared to harvesting in shaping spawning ground distribution in this major fish stock in the North Atlantic Ocean.

## Graphical abstract

Climate warming and harvesting affect the dynamics of species across the globe through a multitude of mechanisms, including distribution changes of spawning fish. For the Northeast Arctic cod, two major hypotheses have been put forward to explain these changes: climate and harvesting. Here, we evaluate these hypotheses by combining two sources of modern data: economic data from the Norwegian fisheries and genetically determined individual fish sampled at the spawning grounds. Our results indicate that demographic size truncation due to harvesting is currently not the dominating factor in shaping spawning ground distribution, but our results provide support for the climate hypothesis.

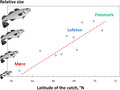

## 1 INTRODUCTION

Many animal species, including birds, mammals, and fish, undertake extensive annual migrations, often to optimize reproductive success. In marine fish, spawning grounds are often spatially separated from feeding and nursery grounds, such as in migration triangle systems (Harden‐Jones, 1968; Secor, 2002). Migration between feeding and spawning grounds is costly because of the energy used to cover the distance (Alexander, 1998). There may also be indirect costs associated with migration, for example lost feeding opportunity due to the time spent on the migration. On evolutionary timescales, the cost of migration has to be balanced by some benefit, which could include direct benefit for the migrating individuals, such as lower risks of predation or disease (Buehler & Piersma, 2008). The benefit may also act through the offspring (i.e., a parental‐offspring trade‐off, Lack, 1954). Offspring benefits associated with the parental migration may include increased survival (Opdal, Vikebø, & Fiksen, 2011) and/or faster growth (Färber, Durant, Vindenes, & Langangen, 2018; Langangen, Ottersen, Ciannelli, Vikebø, & Stige, 2016). Changes in the costs and benefits associated with distinct spawning grounds over time may lead to changes in the distribution of spawning fish. It remains, however, unclear which mechanisms are quantitatively important in causing distribution changes, and this knowledge gap has important ramifications that may impede effective spatially explicit management of fish populations.

**Figure 1:**
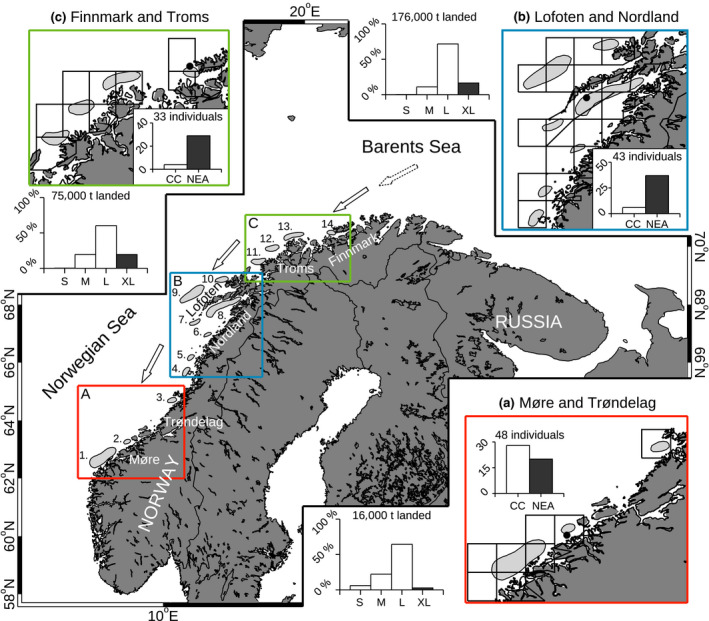
Shows the study area, including the spawning grounds of Northeast Arctic (NEA) cod along the Norwegian coast (gray shaded polygons, numbered 1–14, Sundby & Nakken, 2008). Detailed maps of the three regions ([a] Møre and Trøndelag, [b] Lofoten and Nordland, and [c] Finnmark and Troms) are shown with indication of the reporting areas (solid black lines, Supporting Information Table S1 for a summary of the identification numbers as used by the Norwegian Directorate of Fisheries) that were used in this analysis. A summary of the reported data on cod (2008–2016), including total landings from the spawning grounds in the spawning season in metric tons (t) separated into size classes (S, M, L, XL, Materials and Methods for details) is shown adjacent to each regional map (a–c). The reported weight in the XL class (dark gray box) relative to the total landings is used as a proxy for size of the spawners on the spawning ground. In addition, the number of cod individuals used for genetic determination and the estimated distribution of cod of local origin (coastal cod, CC) and migratory cod (NEA, dark gray box) are shown inside the regional maps. These data were sampled from three different locations on the spawning grounds in Møre, Lofoten, and Finnmark (Black circles) in 2014. Arrows indicate the general direction of the spawning migration from the feeding grounds in the Barents Sea to the spawning grounds. The dotted outline of the arrow indicates that the starting location of the migration is in general unknown

Several potential drivers for species distributions have been suggested, including geographical attachment, environmental conditions, density dependence, demographic structure, and species interactions (Planque, Loots, Petitgas, Lindstrøm, & Vaz, 2011). Quantifying the relative importance of these drivers of fish distribution is essential for our understanding of marine ecosystem dynamics and for a healthy management of marine resources. More specifically for the Northeast Arctic (NEA) stock of Atlantic cod (*Gadus morhua*), it has been documented that the spawning intensity around the main spawning grounds in the Lofoten area (Figure 1) has been fairly stable over time, but spawning in the northern (Finnmark region, Figure 1) and the southern (Møre region, Figure 1) parts of the distribution has been more variable over time (Sundby & Nakken, 2008). These relatively large changes at the fringes of the distribution have led to changes in the mean location of spawning over time. Two hypotheses for explaining the observed changes in the distribution on the spawning grounds have been derived from historic data from before the mid‐1970s: climate warming (Sundby & Nakken, 2008) and harvesting (Opdal, 2010; Opdal & Jørgensen, 2015). First, the costs and benefits associated with the individual spawning ground may change over time due to climate change. For example, climate may affect the spawning distribution directly due to, for example, temperature constraints at the spawning grounds or shifts in the feeding ground distribution potentially caused by climate driven changes in prey distribution (c.f., Fossheim et al., 2015). Such mechanisms may lead to variable use of the spawning grounds on long timescales (Sundby & Nakken, 2008). Note that there is no explicit assumption about variable migration distance with size of the spawners with such a mechanism. From now on, we denote this mechanism the *climate hypothesis*. Second, the distinct spawning grounds may be associated with different energetic costs related to the migration distance. Jørgensen, Dunlop, Opdal, and Fiksen (2008) illustrate how optimal migration distance in NEA cod may be size dependent; large fish are able to migrate farther compared to small fish due to higher energy reserves. Hence, a positive relationship between migration distance and size of the spawners is expected based on this mechanism (Jørgensen et al., 2008). Size of spawners has, in turn, been reported to decrease under high fishing pressure for many heavily exploited fish stocks, including NEA cod (Berkeley, Hixon, Larson, & Love, 2004; Law, 2000; Ottersen, 2008). For the NEA cod, the size and age in the spawning stock decreased from the 1950s to the 1990s (Jørgensen, 1990), potentially caused by the introduction of trawl fisheries at the feeding grounds in the Barents Sea in the first half of the 20th century (Godø, 2003) that primarily target larger individuals and generally are associated with a high fishing mortality. With this theory as a basis, changes in the size structure of the stock have been associated with the variations in the use of spawning grounds over time in NEA cod (Opdal, 2010; Opdal & Jørgensen, 2015). From now on, we denote this the *size truncation hypothesis*. The relative roles of climate and demography in shaping the observed time trend in spawning ground use have been the subject of a scientific debate (Opdal, 2010; Opdal & Jørgensen, 2015, 2016; Sundby, 2015; Sundby & Nakken, 2008), as analyses of two different historic data sets (roe landings [1900–1976] and commercial catches [1866–1969]) gave different results. Recently, over the last two decades, there have been two major changes in the system: increased temperature and a recovering stock biomass and demographic structure (Kjesbu et al., 2014). Here, we follow the recommendation by Opdal and Jørgensen (2015) and investigate how the increased temperature and the recent recovery of the stock observed over the last two decades may affect spawning ground distributions.

## 2 MATERIALS AND METHODS

### 2.1 Study system

The Barents Sea, situated to the north of Norway and northwest of Russia (Figure 1), is a shallow shelf sea that sustains a productive ecosystem, including the large and economically important NEA stock of cod. The NEA cod matures at around age 7–11 (Jørgensen, 1990) and undertakes an annual extensive winter/spring spawning migration from the feeding grounds in the Barents Sea southwards to the coast of Norway (Figure 1). The spawning season lasts from mid‐February to early May with highest activity in March and April (Pedersen, 1984). The timing of the peak and the duration of the spawning have been relatively stable over time (Pedersen, 1984) and are roughly invariant between the Lofoten districts in Norway around 68°N latitude and the Møre districts at about 63°N latitude (Bergstad, Jørgensen, & Dragesund, 1987; Godø & Sunnanå, 1984), but spawning tends to peak slightly later in Finnmark around 70°N latitude (Sundby & Bratland, 1987). Pelagic eggs are released and fertilized at the spawning grounds. The eggs develop into larvae and then juveniles, while drifting with the Norwegian coastal current toward the juvenile nursing areas in the Barents Sea (Ellertsen, Fossum, Solemdal, & Sundby, 1989). For more details on the NEA cod spawning and early life dynamics, see reviews by for example Bergstad et al. (1987) and Ottersen et al. (2014).

### 2.2 Data

To investigate how size of spawning NEA cod varies geographically, we analyzed two data sets on cod size at the spawning grounds.

First, we used information from commercial landing tickets, which are mandatorily filled out at the dockside by the fishermen. Landing ticket information for the landings from boats larger than 11 m fishing cod in Norway in the period 2000–2016 was obtained from the Norwegian Directorate of Fisheries (www.fiskeridir.no). For the analysis of how the size of spawning fish varies between the spawning grounds, we used the years 2008–2016. These years were selected because of an additional reporting class, “very large cod” (XL), included in the reporting from 2008 and onwards. The exact size of cod reported as XL varied in time, with cod (gutted and beheaded) larger than 5.0, 6.5, and 6.0 kg reported as XL in 2008–2009, 2010–2011, and 2012–2016, respectively. This data subset constitutes about 3.9 million entries, with information on, among others, species, product state (e.g., gutted and beheaded fish or full fish, or byproducts such as liver or roe), weight class of landed fish, total weight of landing, main catch area, gear type used, landing time, boat size, ship ID. From this data set, we extracted the cod entries (about 1.25 million entries) that originated from the spawning season (March and April, 32% of the cod entries) from known NEA cod spawning locations (Sundby & Nakken, 2008 and Figure 1, 57% of the remaining entries). We focused the analysis on the two main months of the spawning season and on geographically known spawning locations to minimize the potential for errors caused by fish that are still migrating to a spawning ground or by non‐spawning fish. Furthermore, we focused on gutted and beheaded cod, as this product state is the most abundantly reported (about 54% of all cod entries). Note that we performed a similar analysis on the other product states and obtained similar results (Supporting Information). The remaining entries were assigned to individual spawning grounds from Møre in the South to Finnmark in the North (Figure 1). We aggregated the two southern spawning grounds in the Møre region (spawning ground 1 and 2 in Figure 1) because there were few data available for one of the spawning grounds (spawning ground 2). Similarly, we aggregated the two southern spawning grounds in the Nordland region (spawning ground 4 and 5 in Figure 1) due to few entries from one of these spawning grounds (spawning ground 4). Also, in the Troms region (Figure 1), multiple spawning grounds are overlapping within the same grid cell, making it difficult to unambiguously assign the reported catch to individual spawning grounds. As a result, we aggregated the three southern spawning grounds in this region (spawning grounds 11–13 in Figure 1). In total, we obtained 10 data groups representing spawning grounds.

Because of variable fishing gear use along the coast of Norway and because fishing gear typically harvest size selectively (Diekert, Hjermann, Nævdal, & Stenseth, 2010), we investigated reported size based on different gear types. The gear types that were abundantly present in the data set included gillnets (here taken as the two reporting classes “gillnet” and “undefined net”), line fishing (taken as “autoline,” “floatingline,” “juksa/pilk,” and “other lines”), seine fishing, and trawl fishing. However, due to strong geographical variations in gear use for line fishing and seine fishing (<1% of the reports coming from the spawning grounds south of 67°N) and in general few data and many spawning grounds without data for the trawl fishery, we focused the analysis on the gillnet fishery (consisting of about 70% of the entries, Supporting Information Figure S4, in total about 85,000 entries) that was well distributed across the whole geographic range (Supporting Information, for a similar analysis based on the line fishing). In total, these entries represent about 250,000 tons of landed cod (Figure 1).

Landed cod was sold for a price based on the size of the individual fish (as well as other quality measures such as damage to the fish, etc.). Due to this size‐dependent pricing, the landings were reported size class specific (four classes: small fish [S] < 1 kg, medium fish [M] between 1 and 2.5 kg, large fish [L] between 2.5 and very large class limit, very large fish [XL] > very large class limit). The very large class limit varied over time, with a limit of 5.0 kg in 2008–2009, 6.5 kg in 2010–2011, and 6.0 kg in 2012–2016; all weights are for gutted and beheaded fish. We used the spawning ground‐specific landed weight from the XL cod class (we_large_) relative to the total landed weight (we_total_) as a proxy for very large fish. Note that the number of individual fish and the size of individual fish within the size classes are confounded in this proxy (e.g., two fish each of 7 kg will be reported equal to one fish of 14 kg). This will, however, not likely affect the analysis, as both these aspects are relevant measures of size.

**Figure 2:**
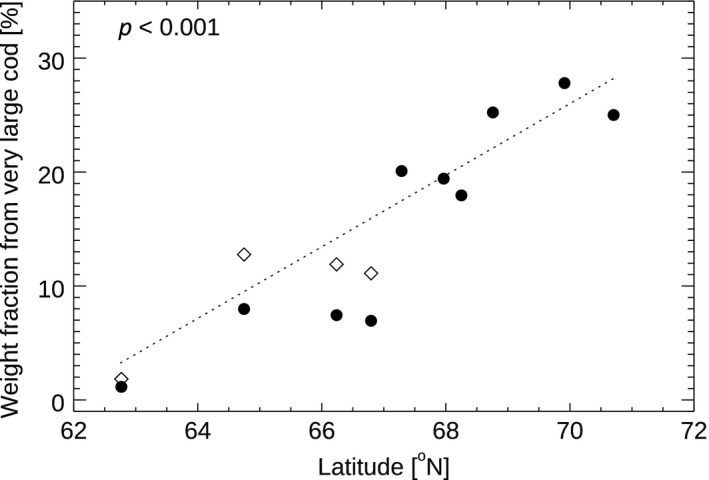
Shows the spawning ground‐specific weight fraction (black filled circles*,* weight in very large class divided by total weight) calculated from landing tickets from the years 2008–2016. In addition, the weight fraction corrected for coastal cod (open diamonds) is shown. Dotted line indicates the linear regression for the corrected data showing a significant (*p* < 0.001) increase in weight fraction with latitude

We took into account that Norwegian coastal cod, which is typically of local origin and does not undertake extensive migration to the spawning grounds (Jakobsen, 1987), is present in the catch at the NEA cod spawning grounds (Berg & Albert, 2003; ICES, 2017). The fraction of coastal cod in the catch south of 67°N was, for example, reported to be around 60% in the two first quarters in 2013–2016 (ICES, 2015, table 2.4 for 2013–2014, ICES, 2017, table 2.4 for 2015–2016), while only about 10% of the catch north of 67°N was coastal cod. This could potentially bias our results since the coastal cod is smaller at age and matures almost a year earlier than the NEA cod (Berg & Albert, 2003), which could artificially reduce the size of spawners in areas where the coastal cod is more abundant. We correct for this potential bias by calculating the size distribution of coastal cod for the period 2008–2016 and comparing this with the size distribution of NEA cod. This calculation was done using data from ICES (ICES, 2017), tables 3.9, 3.11, and 3.21 for NEA cod and tables 2.6, 2.8, and 2.10 for coastal cod). The results of this test indicate that a multiplicative correction of 1.6 in the weight fraction (we_large_/we_total_) for the spawning grounds south of 67°N would be suitable (Supporting Information for further details). We apply such a correction to the data (Figure 2) and aggregate the weight fraction over years. The weight fraction was then regressed against latitude.

Second, to further address the possible impact of size estimation due to the potential cryptic presence of coastal cod in our data, we analyzed a second data set of individual cod caught with gillnet or line fisheries at the spawning grounds in Møre, Lofoten, and Finnmark during the spawning season of 2014. The cod were caught by a 180 mm mesh gillnet at Møre, and with line at Lofoten and Finnmark (Supporting Information Tables S2–S5 for detailed individual information and the Supplementary Results for a test of gear selectivity). Only those individuals were selected that were genetically determined to be NEA cod (Supporting Information Table S2) through analysis of genotype data from single nucleotide polymorphisms (SNP) selected from a 12 k SNP chip (The Cod SNP Consortium, in preparation; but see Berg et al., 2015; Berg et al., 2016; Berg et al., 2017, Kirubakaran et al., 2016; Barth et al., 2017; for further details see also Supporting Information and Figure S1). These SNPs were specifically selected to genotype four mega‐base scale polymorphic regions with high linkage disequilibrium (Supporting Information and Table S2 for further details)—most likely genomic inversions—that segregate with a distinct geographical distribution among cod populations (Barth et al., 2017; Berg et al., 2016; Kirubakaran et al., 2016; Sodeland et al., 2016). Because the inversions are located on different chromosomes, independence between loci can be assumed. It is therefore straightforward to calculate the overall probability of obtaining a composite inversion genotype as a measure of an individual's affinity toward either the NEA or coastal population (Star et al., 2017). Calculating this overall probability, we compare the size of those individuals from Møre, Lofoten, and Finnmark, that have a >97.5% probability of belonging to the NEA population (being less conservative by changing the cutoff to 90% did not change the main result, that is, that fish are smaller toward the southern part), excluding any suspect coastal individuals (Supporting Information Tables S2–S5). We performed a one‐way analysis of variance by using the “*aov*” function in r to compare the log‐transformed length of the spawning NEA cod from the three spawning grounds. Furthermore, we post‐hoc calculated the Tukey Honest Significant Differences by using the “*TukeyHSD*” function in r to evaluate size differences between individual pairs of spawning grounds.

Moreover, to elaborate on the mechanisms driving changes in spawning ground use, we constructed a proxy for spawning location for each year from 2000 to 2016. As a proxy for spawning location, we used mean latitude of landed cod weight from the landing tickets data. We restricted the data to the reported total landed weight of cod from the known spawning locations in the spawning season (see above for details). We correlated the time series of mean latitude of spawning (*mlat*) with relevant covariates, such as mean weight of spawners and the temperature. As a temperature proxy for the cod migration and spawning period, we used the temperature measured at the Kola transect (Tereshchenko, 1996). For this comparison, we used the winter–spring temperature calculated as the mean temperature from January–April the year of spawning from the upper 200 m depth (stations 3–7, representing temperatures in the Atlantic water masses). We calculated the biomass weighted mean weight of spawners (MW). The MW*_i_* was calculated on a yearly basis (*i*), using the weight at age (*W_a,i_*), numbers at age (*N_a,i_*), and proportion mature at age (*M_a,i_*) as reported by ICES (ICES, 2017):

$${\text{MW}}_{i}=\frac{\sum_{a}W_{a,i}^{2}N_{a,i}M_{a,i}}{\sum_{a}W_{a,i}N_{a,i}M_{a,i}}$$

Finally, we correlated the two explanatory variables separately with the *mlat*. To account for autocorrelation in the time series, we followed the method suggested by Pyper and Peterman (1998), which accounts for the effective degrees of freedom in calculating the significance of the correlation**.**

## 3 RESULTS

Based on the reported Norwegian catch data from 2008 to 2016, we constructed a proxy for the size of spawning cod on individual spawning grounds (Figure 1) by calculating the weight fraction of landings from very large cod (“XL” in Figure 1) relative to total cod landings. This proxy shows an increasing trend in size toward the northern end of the spawning distribution (Figure 2). The positive association between the latitude of the spawning ground and the size of spawners is statistically significant (*p* < 0.001, *R* ^2^ = 0.85, *N* = 10), that is, the size of the spawning NEA cod decreases with increasing distance from the feeding grounds in the Barents Sea.

**Figure 3:**
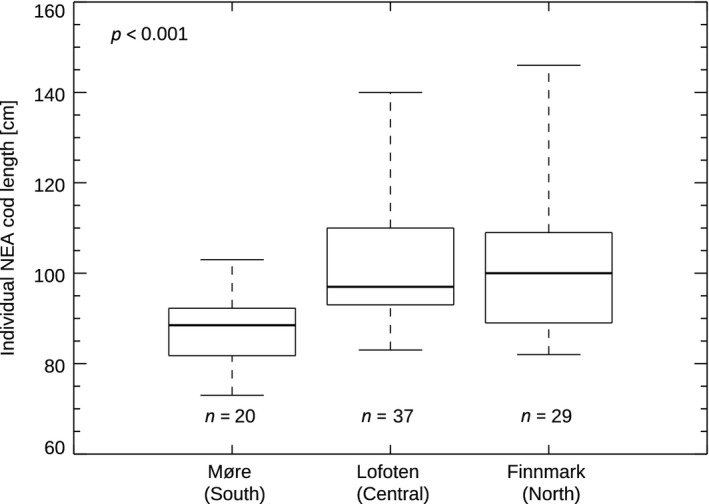
Box plot (showing 0%, 25%, 50%, 75%, and 100% quantiles of the data) indicating the observed length of the individual data on Northeast Arctic (NEA) cod caught in the spawning season in Møre, Lofoten and Finnmark in 2014. The number of individual fish determined as NEA cod is given directly below each box plot and the *p* refers to the one‐way ANOVA test, indicating that NEA cod from Møre are smaller compared to NEA cod in Lofoten and Finnmark

Furthermore, to separate between the migratory NEA cod and the coastal cod of local origin, we performed an analysis of individual genotype data (sampled on the spawning grounds in 2014; Figure 1) from single nucleotide polymorphisms (SNPs). We found that more than 80% of the individuals could be assigned as NEA cod with more than 97.5% probability in the two northern districts (Lofoten: 37 out of 43 individuals and Finnmark: 29 out of 33 individuals), while only around 40% of the individuals caught in the southern region (Møre: 20 out of 48 individuals) were NEA cod. We performed a one‐way ANOVA test on the log_e_‐transformed length of the NEA cod from the three different spawning grounds and found a significant difference in size among the spawning grounds (*p* < 0.001, Figure 3). To corroborate this further, we performed a post hoc Tukey honest significant differences test, indicating that the mean size of the spawners in the southern spawning ground (Møre) was smaller than the mean size of the spawners in the two more northern spawning grounds (Lofoten and Finnmark, *p* < 0.01, Figure 3), while the size of the spawning NEA cod on the two northern spawning grounds did not differ significantly.

**Figure 4:**
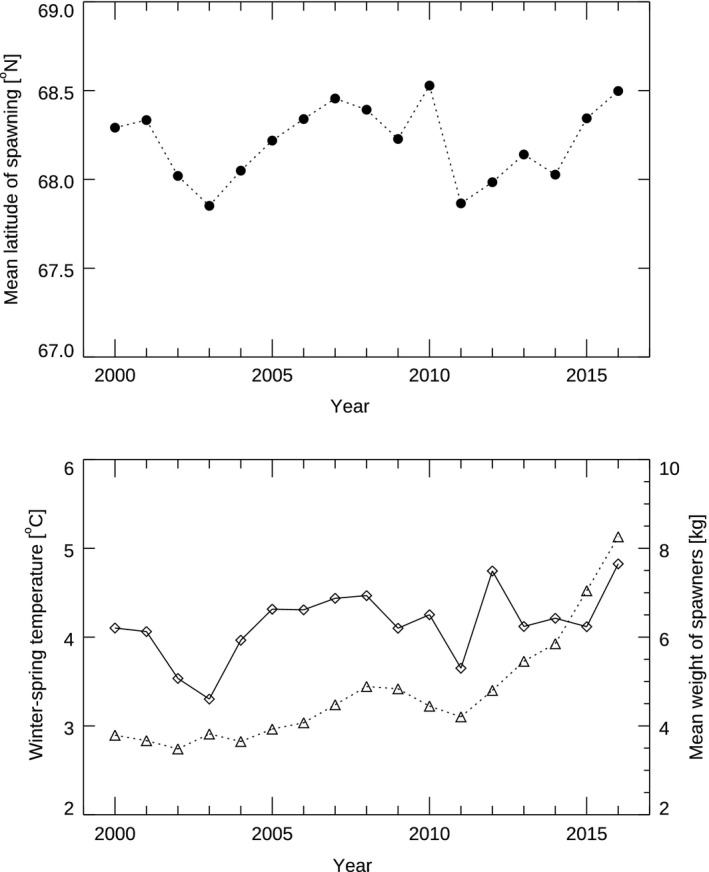
Shows the mean latitude of spawning (black solid circles, upper panel) as calculated from the landing ticket data. Furthermore, the winter‐spring temperatures as measured at the Kola section are shown (open diamonds, lower panel) together with the mean weight of spawners (open triangles, lower panel)

The calculated mean latitude of spawning is shown in Figure 4, together with the Kola temperature and the mean weight of spawners. We found that the Kola‐temperature was positively and significantly correlated with the mean latitude of spawning (product–moment correlation coefficient: *r* = 0.62, effective degrees of freedom: *df* = 12.4 and *p* = 0.016) and that mean weight was positively but not significantly correlated (product‐moment correlation coefficient: *r* = 0.35, effective degrees of freedom: *df* = 11.5 and *p* = 0.23). Note that a test of a temperature proxy for the feeding season prior to the migration did not correlate significantly with the mean latitude of spawning (product‐moment correlation coefficient: *r* = 0.24, effective degrees of freedom: *df* = 15.9 and *p* > 0.1).

## 4 DISCUSSION

Our analysis of the size of the spawners at different spawning grounds shows an increasing size of spawners with increasing latitude. The correlation remains significantly positive also after correcting for the higher fraction of the smaller sized non‐migratory coastal cod in the southern spawning grounds (Figure 2). To substantiate our finding on the size differences between the spawning grounds, we investigated individuals genetically determined to be NEA cod. We indeed find that the NEA cod caught in the northern areas (Lofoten and Finnmark) during the spawning season were bigger compared to NEA cod caught in the southern area (Møre; Figure 3). The observed pattern in size between the spawning grounds are opposite of the pattern predicted by the *size truncation hypothesis*. Moreover, our analysis of the changes in spawning location over time suggests more northern spawning in years with warm winter‐spring temperatures between 2000 and 2016. These results give support for climate as an important factor in shaping the spawning ground distribution of NEA cod in the recent period.

We interpret the observed correlation of size of spawners and latitude as strong indication that the size of the spawning fish is decreasing with the distance from the feeding ground. This is somewhat surprising as a meta‐study based on 23 fish species (including cod) has previously shown a positive correlation between migration distance and fish size at the species level (Roff, 1988). Taken together with the positive correlation between the spawning location and the winter‐spring temperature over time, we conclude that our results are not supporting the *size truncation hypothesis* as the main factor determining the spawning location of NEA cod in recent decades. According to the *size truncation hypothesis*, the size of spawners is expected to increase—rather than decrease (Figures 2 and 3)—with distance from the feeding grounds (potentially about 10 cm larger in Møre compared to Lofoten, Jørgensen et al., 2008) and we would expect a negative correlation between, for example, mean weight of spawners and spawning location. Note that in the most recent years (2015 and 2016), there has been an increase in age at maturity (ICES, 2017), which has contributed to the increase in mean weight of spawners (Figure 4), without a clear response in the spawning location in these years.

While the observed positive correlation between size of spawners and latitude of the spawning ground is not directly supporting the *climate hypothesis* (Sundby & Nakken, 2008), such a trend is conceivable within this framework. For example, if there is subpopulation structure with defined size structures and the subpopulations respond differently to climate warming, one could potentially observe a decreasing size with decreasing latitude (Sundby & Nakken, 2008). The temporal pattern found, with more northerly spawning in years with warm compared to cold winter‐spring temperatures, could, for example, be related to the feeding areas in the Barents Sea extending farther north and eastward in warm years (e.g., Renaud et al., 2011). While we do not know why the *size truncation hypothesis* is not supported by modern data, potential reasons include lacking contrast in the size structure over the last two decades and irreversible changes in spawning strategies due to loss of social learning or evolutionary changes (Opdal & Jørgensen, 2015).

Although we clearly demonstrate that spawner size decreases with decreasing latitude (a proxy for north–south distance from the feeding grounds) for NEA cod, our methods cannot unravel the mechanisms behind this pattern. One possible mechanism for the decreasing size toward the south may be size structure on the feeding grounds. However, exactly how the fish would structure on the feeding grounds according to size or age is unclear. Young cod (age 7 and younger) tend to be distributed more north and eastward with increasing age inside the Barents Sea in the autumn, as indicated by the age‐specific distribution maps shown in Johansen, Johannesen, Michalsen, Aglen, and Fotland (2013). But we note that cod typically follow the southwards spawning migration of capelin and will thus often aggregate in the southern Barents Sea in the winter (Yaragina, Aglen, & Sokolov, 2011). Overall, it is challenging to measure when and where the spawning migration starts, and hence the actual migration distance (Sundby, 2015). Nevertheless, we do not expect the interannual variability in the center of gravity of the feeding ground distribution to significantly impact our main result, the increasing size of spawners with latitude, as it is based on the average size over several years (2008–2016). It is also unclear how distributions at spawning and feeding grounds are linked. Results of tagging experiments suggest that fish spawning in Møre overlap with fish spawning in Lofoten at the feeding grounds in the Barents Sea. But there are some indications that the fish spawning in Møre are distributed more toward the western parts of the Barents Sea (Godø, 1984). Another potential mechanism for the decreasing size toward the south, as mentioned above, could be subpopulation structure (Sundby & Nakken, 2008). If, for example, different subpopulations have distinct size structure, the observed patterns in size with distance from the feeding grounds could be a result of these distinct size distributions. Finally, density‐dependent competition at the spawning grounds (Höffle et al., 2014) may be an important mechanism. Large fish start their spawning migrations earlier (Bergstad et al., 1987) and may potentially occupy the best spots, forcing smaller and late‐arriving individuals to spawn further south.

Whether loss of southern spawning grounds results in loss of genetic or behavioral diversity depends on whether fish home to the same location. Even though there are examples where Atlantic cod that undertake long‐distance feeding migrations may home to a specific spawning ground in consecutive years (Svedäng, Righton, & Jonsson, 2007), not all migratory individuals are “accurate homers” (Robichaud & Rose, 2004). At present, it is unclear to what extent the different spawning areas for NEA cod along the Norwegian coast is linked to natal homing individuals. Such homing could be sustained over time by, for example, genetics, memory, and social learning (cf. Rogers, Salomon, Connors, & Krkošek, 2018). Hence, it is unclear whether a potential loss of southern spawning areas would also result in a change or loss in genetic population structure or socially learned spawning strategies (De Luca, Mariani, MacKenzie, & Marsili, 2014).

While the landing ticket data rely on a sample of roughly 85,000 observations, the sample size of the genetic data is much smaller (*N* = 124) due to the high costs of acquiring such data. Despite this low sample size, we find a clear, statistically significant pattern (*p* < 0.001) that fully agrees with the results from the analysis of the commercial landing tickets data. These landing ticket data, however, do not include information on geographical differences in fishing practices. If the fishermen use gillnets with different mesh widths at different spawning grounds, that is, actively selecting for different fish size, this could potentially bias the size of the caught fish. We have therefore tested if gear selectivity could be the main reason for the trend by analyzing available data on gear use from a subset of the fishing vessels (Supporting Information). The results of this test indicate that gear selectivity can explain only a fraction of the observed trend (Supporting Information Figure S7), and that this selectivity does not affect our conclusion on differences in the size distribution at the spawning grounds.

When fishing in different regions affects the size distribution of the fish population differently, as is often the case with migratory fish stocks, fisheries management must be spatially explicit to be successful (Stelzenmüller, Ellis, & Rogers, 2010). Traditionally, fishermen target spawning aggregations because the high density of available fish lowers the cost of harvesting (de Mitcheson & Erisman, 2011; Erisman et al., 2012), and because large fish tend to yield a better price (per kg) compared to small fish (Zimmermann & Heino, 2013). Fisheries tend to track shifts in the fish distribution, but typically do so with a significant time lag (Pinsky & Fogarty, 2012). Importantly, technological or administrative constraints may limit the adaptability of fishing effort to changes in the spatial distribution, leading to unintended and undesired consequences. Prolonged periods of spatial mismatch between fishing pressure and fish stock may endanger the very existence of local fish stocks, especially if there are unobserved substructures in the population (Sterner, 2007). A better understanding of which factors drive changes in fish distribution may allow for a more proactive spatially explicit management of the fish stocks. Moreover, knowledge on the variation in fish size across spawning grounds reveals possible links between the geographic distribution of fishing and size composition of catches. A broad size distribution of a fish population is a politically mandated aim of the EU's fisheries policy (European Commission, 2008) because it is in general considered to be a sign of a healthy stock. Although there is some debate, for example, to what extent a diverse size distribution contributes to increased recruitment (Hixon, Johnson, & Sogard, 2014; Stige et al., 2017), it is clearly important to evaluate the effect of how fishing on a geographically size structured spawning population affects the whole population structure and dynamics.

We have illustrated how a combination of data sources, one large economic data set based on dock side landing reports and a data set based on genetic analysis of individual spawners, can be used to evaluate ecological hypotheses that have large socioeconomic ramifications. Our results underscore the importance of testing such hypotheses with different data sets. In particular, our results indicate that *demographic size truncation* due to fisheries is currently not the dominating factor in shaping spawning migration and the distribution at the spawning grounds for NEA cod. Our results instead provide support for the *climate hypothesis* (Sundby & Nakken, 2008). However, one should not forget that climate and demography are not the only drivers explaining the variation in fish distributions (Thorson, Ianelli, & Kotwicki, 2017). Future research on this topic is urgently needed to investigate the impact of other potential drivers, such as density dependence, geographic attachment, and species interactions (Planque et al., 2011). Successful management of fisheries relies on spatial policies that are aligned to the underlying ecological facts.
