# Supplementary material for: Ticket to spawn: Combining economic and genetic data to evaluate the effect of climate and demographic structure on spawning distribution in Atlantic cod
Source: Glob Chang Biol. 2018 Oct 30;25(1):134–43. doi: 10.1111/gcb.14474 (PMC7379705; doi:10.1111/gcb.14474)
Supplement: Supplementary file 1 [file GCB-25-134-s001.docx]

**Supplementary information for “Ticket to spawn: Combining economic and genetic data to evaluate the effect of climate and demographic structure on spawning distribution in cod“**

**Area codes for the areas considered cod spawning grounds**

Table S1 shows the area codes used by the Norwegian Directorate of Fisheries for the different spawning grounds.

Table S 1 shows an overview of the catch area IDs used to assign catch to different spawning grounds (see Figure 1 in main text).

| Spg.nr | 1-2 | 3 | 4-5 | 6 | 7 | 8 | 9 | 10 | 11-13 | 14 |
| --- | --- | --- | --- | --- | --- | --- | --- | --- | --- | --- |
| Catch area id | 07-07 | 06-12 | 06-27 | 00-05 | 00-03 | 00-48 | 05-18 | 05-19 | 04-01 | 04-11 |
|  | 07-15 |  | 06-23 | 06-31 | 00-04 | 00-47 | 05-13 | 05-20 | 04-02 | 04-04 |
|  | 07-05 |  | 06-33 | 06-32 | 00-49 | 00-46 | 05-14 |  | 05-35 | 04-05 |
|  | 07-06 |  |  |  |  | 00-44 |  |  | 05-30 |  |
|  | 07-33 |  |  |  |  | 00-11 |  |  | 05-31 |  |
|  | 07-31 |  |  |  |  | 00-10 |  |  | 05-29 |  |
|  | 07-19 |  |  |  |  | 00-50 |  |  | 05-25 |  |

**The underlying data for the genetic determination of individual fish**

Frist, all specimens were genotyped using a set of 48 SNPs on a Fluidigm platform that was based on results from a larger data set using a 12 k SNP chip (The Cod SNP Consortium, in preparation; but see Berg *et al*., 2015; Berg *et al.*, 2016; Kirubakaran *et al.*, 2016; Sodeland *et al.*, 2016; Barth *et al.*, 2017; Berg *et al.*, 2017) by a Bayesian analysis for differentiation outliers (Foll & Gaggiotti 2008). These 48 SNPs were specifically selected to distinguish between NEA cod and coastal cod, including the four mega-base scale genomic inversions that segregate with a distinct geographical distribution among cod populations (Berg *et al.*, 2016; Kirubakaran *et al.*, 2016; Sodeland *et al.*, 2016; Barth *et al.*, 2017). This analysis was performed with a genotype data set for 24 individuals known to be NEA cod and 24 individuals known to be coastal cod according to the ecotype classification of Berg et al. (2016), as well as 527 coastal individuals from the Eastern North Atlantic south of the range of NEA cod (Barth *et al*. 2017; see Figure S1).


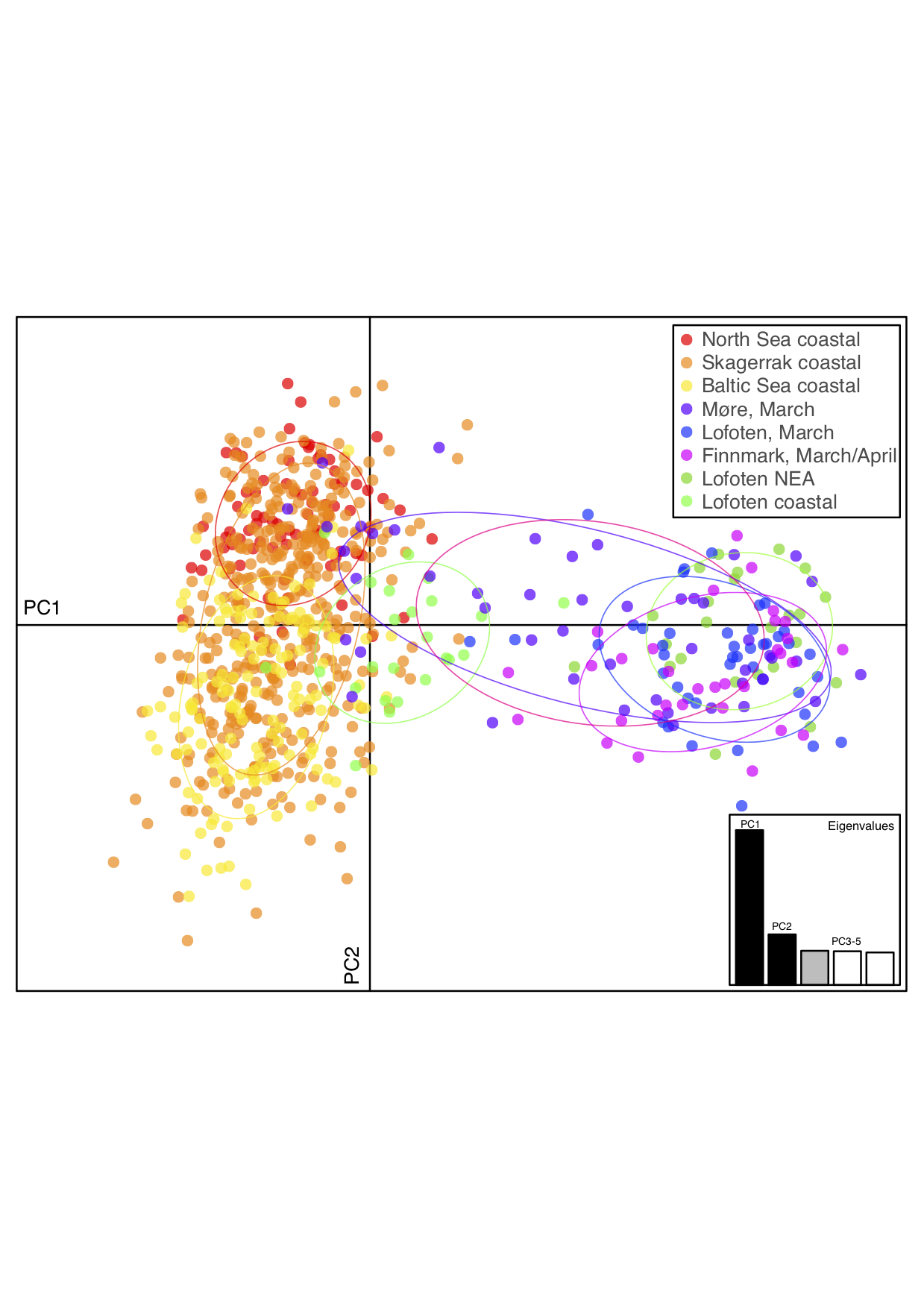
**Figure S1: Principal component analysis based on the selected 48 SNPs, including individuals genotyped on the 12k SNP (North Sea coastal, Skagerrak coastal, Baltic Sea coastal, Lofoten NEA, and Lofoten coastal), as well as individuals only genotyped based on the 48 SNPs (Møre, March, Lofoten, March, Finnmark, March/April).**

Second, for a proper classification of NEA cod and costal cod we calculated the probability of obtaining an inversion genotype, which follows a binomial distribution given the underlying allele frequency in a population (Star *et al.*, 2017). This was done by using the selected SNPs on each of the inversions ((LG01: ss1712296486, LG02: ss 1712296037, LG07: ss1712303816, LG12: ss 1712300137) that were included in the set of 48 SNPs (see Table S2).

Table S2 shows the relative allele frequency distribution assumed for coastal cod and NEA cod (Star *et al*., 2017). Furthermore, an overview of the individually determined fish caught on the spawning grounds in the Møre, Lofoten and Finnmark regions in 2014 are shown in tables S3-5.

Table S 2 shows the relative allele frequencies of four inversions in NEA and coastal cod populations.

|  | NEA cod | | | | Coastal cod | | | |
| --- | --- | --- | --- | --- | --- | --- | --- | --- |
|  | LG01 | LG02 | LG07 | LG12 | LG01 | LG02 | LG07 | LG12 |
| AA | 0.0278 | 0.00694 | 0.00391 | 0 | 0.927 | 0.25 | 0.420 | 0.0219 |
| AB | 0.278 | 0.153 | 0.117 | 0.05 | 0.0713 | 0.5 | 0.456 | 0.252 |
| BB | 0.694 | 0.840 | 0.879 | 0.95 | 0.00137 | 0.25 | 0.124 | 0.726 |

Table S 3 shows the individual data obtained in the Møre district 24^th^-26^th^ March 2014.

| Sample | LG01 | LG02 | LG07 | LG12 | Prob. NEAC | Prob. Coastal | Determination | Length (cm) |
| --- | --- | --- | --- | --- | --- | --- | --- | --- |
| 01 | AB | BB | BB | BB | 0.99 | 0.01 | NEAC | 75 |
| 02 | AA | AB | AB | BB | 0.00 | 1.00 | CC | 84 |
| 03 | BB | BB | BB | BB | 1.00 | 0.00 | NEAC | 92 |
| 04 | AA | AB | AB | AA | 0.00 | 1.00 | CC | 85 |
| 05 | AA | AB | AA | BB | 0.00 | 1.00 | CC | 79 |
| 06 | BB | BB | BB | AB | 1.00 | 0.00 | NEAC | 76 |
| 07 | AA | BB | BB | BB | 0.48 | 0.52 | CC | 82 |
| 08 | AA | AB | AB | BB | 0.00 | 1.00 | CC | 80 |
| 09 | AB | AB | BB | BB | 0.92 | 0.08 | CC | 83 |
| 10 | AB | AB | AB | BB | 0.29 | 0.71 | CC | 76 |
| 11 | AA | AB | AA | AA | 0.00 | 1.00 | CC | 76 |
| 12 | AA | AB | AA | BB | 0.00 | 1.00 | CC | 89 |
| 13 | AA | BB | AA | BB | 0.00 | 1.00 | CC | 76 |
| 14 | AA | AB | AA | AB | 0.00 | 1.00 | CC | 71 |
| 15 | AA | AB | AB | BB | 0.00 | 1.00 | CC | 85 |
| 16 | BB | BB | BB | BB | 1.00 | 0.00 | NEAC | 73 |
| 17 | BB | BB | BB | BB | 1.00 | 0.00 | NEAC | 80 |
| 18 | BB | BB | BB | BB | 1.00 | 0.00 | NEAC | 102 |
| 19 | BB | BB | BB | BB | 1.00 | 0.00 | NEAC | 81 |
| 20 | AA | AB | AB | AB | 0.00 | 1.00 | CC | 79 |
| 21 | AB | BB | BB | BB | 0.99 | 0.01 | NEAC | 102 |
| 22 | AB | AB | AB | AB | 0.06 | 0.94 | CC | 76 |
| 23 | AA | AB | AB | BB | 0.00 | 1.00 | CC | 95 |
| 24 | AA | AB | AB | AB | 0.00 | 1.00 | CC | 66 |
| 25 | BB | BB | BB | BB | 1.00 | 0.00 | NEAC | 88 |
| 26 | AB | AB | AB | BB | 0.29 | 0.71 | CC | 79 |
| 27 | AB | BB | BB | BB | 0.99 | 0.01 | NEAC | 93 |
| 28 | AA | AB | AA | AB | 0.00 | 1.00 | CC | 92 |
| 29 | AA | AB | AB | AB | 0.00 | 1.00 | CC | 80 |
| 30 | AB | BB | AA | BB | 0.14 | 0.86 | CC | 92 |
| 31 | BB | BB | BB | BB | 1.00 | 0.00 | NEAC | 89 |
| 32 | AA | AB | AB | AB | 0.00 | 1.00 | CC | 71 |
| 33 | BB | AB | BB | AB | 1.00 | 0.00 | NEAC | 97 |
| 34 | AA | AB | AA | AB | 0.00 | 1.00 | CC | 94 |
| 35 | AB | AB | BB | BB | 0.92 | 0.08 | CC | 84 |
| 36 | BB | BB | BB | BB | 1.00 | 0.00 | NEAC | 88 |
| 37 | AA | AB | BB | BB | 0.08 | 0.92 | CC | 72 |
| 38 | BB | BB | BB | BB | 1.00 | 0.00 | NEAC | 88 |
| 39 | AB | AB | AB | BB | 0.29 | 0.71 | CC | 84 |
| 40 | BB | BB | BB | BB | 1.00 | 0.00 | NEAC | 103 |
| 41 | AA | AB | AA | BB | 0.00 | 1.00 | CC | 77 |
| 42 | BB | BB | BB | BB | 1.00 | 0.00 | NEAC | 90 |
| 43 | AB | BB | BB | BB | 0.99 | 0.01 | NEAC | 82 |
| 44 | AB | AB | BB | BB | 0.92 | 0.08 | CC | 98 |
| 45 | AA | AB | AB | BB | 0.00 | 1.00 | CC | 70 |
| 46 | BB | BB | BB | BB | 1.00 | 0.00 | NEAC | 89 |
| 47 | BB | BB | BB | BB | 1.00 | 0.00 | NEAC | 92 |
| 48 | BB | BB | BB | BB | 1.00 | 0.00 | NEAC | 83 |

Table S 4 shows the individual data obtained in the Lofoten district 18^th^-19^th^ March 2014

| Sample | LG01 | LG02 | LG07 | LG12 | Prob. NEAC | Prob. Coastal | Determination | Length (cm) |
| --- | --- | --- | --- | --- | --- | --- | --- | --- |
| 01 | AB | AB | AA | BB | 0.01 | 0.99 | CC | 77 |
| 02 | BB | BB | BB | AB | 1.00 | 0.00 | NEAC | 95 |
| 03 | BB | BB | BB | AB | 1.00 | 0.00 | NEAC | 91 |
| 04 | BB | BB | BB | BB | 1.00 | 0.00 | NEAC | 94 |
| 05 | BB | BB | BB | AB | 1.00 | 0.00 | NEAC | 112 |
| 06 | BB | BB | BB | BB | 1.00 | 0.00 | NEAC | 87 |
| 07 | BB | BB | BB | BB | 1.00 | 0.00 | NEAC | 102 |
| 08 | BB | BB | BB | BB | 1.00 | 0.00 | NEAC | 97 |
| 09 | BB | BB | BB | BB | 1.00 | 0.00 | NEAC | 87 |
| 10 | BB | BB | BB | BB | 1.00 | 0.00 | NEAC | 98 |
| 11 | AB | BB | BB | BB | 0.99 | 0.01 | NEAC | 110 |
| 12 | BB | BB | BB | BB | 1.00 | 0.00 | NEAC | 93 |
| 13 | BB | BB | BB | BB | 1.00 | 0.00 | NEAC | 105 |
| 14 | BB | BB | BB | BB | 1.00 | 0.00 | NEAC | 107 |
| 15 | AB | BB | BB | BB | 0.99 | 0.01 | NEAC | 113 |
| 16 | BB | BB | BB | BB | 1.00 | 0.00 | NEAC | 96 |
| 17 | AB | AB | BB | BB | 0.92 | 0.08 | CC | 91 |
| 18 | BB | BB | BB | BB | 1.00 | 0.00 | NEAC | 110 |
| 19 | BB | BB | BB | BB | 1.00 | 0.00 | NEAC | 121 |
| 20 | BB | BB | BB | BB | 1.00 | 0.00 | NEAC | 112 |
| 21 | BB | BB | BB | BB | 1.00 | 0.00 | NEAC | 140 |
| 22 | BB | BB | AB | BB | 1.00 | 0.00 | NEAC | 120 |
| 23 | BB | BB | BB | BB | 1.00 | 0.00 | NEAC | 96 |
| 24 | AB | BB | BB | BB | 0.99 | 0.01 | NEAC | 93 |
| 25 | AB | BB | BB | BB | 0.99 | 0.01 | NEAC | 105 |
| 26 | BB | BB | BB | BB | 1.00 | 0.00 | NEAC | 90 |
| 27 | BB | BB | BB | BB | 1.00 | 0.00 | NEAC | 102 |
| 28 | AA | AB | BB | BB | 0.08 | 0.92 | CC | 120 |
| 29 | AB | BB | BB | BB | 0.99 | 0.01 | NEAC | 87 |
| 30 | AB | BB | BB | AB | 0.95 | 0.05 | CC | 88 |
| 31 | BB | BB | BB | BB | 1.00 | 0.00 | NEAC | 86 |
| 32 | AB | BB | BB | BB | 0.99 | 0.01 | NEAC | 83 |
| 33 | BB | BB | BB | AB | 1.00 | 0.00 | NEAC | 86 |
| 34 | AB | BB | BB | BB | 0.99 | 0.01 | NEAC | 111 |
| 35 | BB | BB | BB | BB | 1.00 | 0.00 | NEAC | 97 |
| 36 | AB | AB | BB | BB | 0.92 | 0.08 | CC | 78 |
| 37 | AB | AB | BB | BB | 0.92 | 0.08 | CC | 94 |
| 38 | BB | BB | BB | BB | 1.00 | 0.00 | NEAC | 92 |
| 39 | BB | BB | BB | BB | 1.00 | 0.00 | NEAC | 117 |
| 40 | AB | BB | BB | BB | 0.99 | 0.01 | NEAC | 96 |
| 41 | BB | BB | BB | BB | 1.00 | 0.00 | NEAC | 124 |
| 42 | BB | BB | BB | AB | 1.00 | 0.00 | NEAC | 97 |
| 43 | BB | BB | BB | BB | 1.00 | 0.00 | NEAC | 100 |

Table S 5 shows the individual data obtained in the Finnmark district 31^th^ March and 3^rd^ April 2014

| Sample | LG01 | LG02 | LG07 | LG12 | Prob. NEAC | Prob. Coastal | Determination | Length (cm) |
| --- | --- | --- | --- | --- | --- | --- | --- | --- |
| 01 | BB | BB | BB | BB | 1.00 | 0.00 | NEAC | 97 |
| 02 | BB | BB | BB | BB | 1.00 | 0.00 | NEAC | 90 |
| 03 | Invalid | Invalid | Invalid | Invalid | 1.00 | 0.00 | NaN | 109 |
| 04 | AB | BB | BB | BB | 0.99 | 0.01 | NEAC | 135 |
| 05 | AB | BB | BB | BB | 0.99 | 0.01 | NEAC | 89 |
| 06 | AB | BB | BB | BB | 0.99 | 0.01 | NEAC | 82 |
| 07 | BB | AB | AB | BB | 0.98 | 0.02 | NEAC | 87 |
| 08 | BB | BB | BB | AB | 1.00 | 0.00 | NEAC | 103 |
| 09 | BB | BB | BB | AB | 1.00 | 0.00 | NEAC | 84 |
| 10 | AB | BB | BB | BB | 0.99 | 0.01 | NEAC | 89 |
| 11 | AA | BB | BB | AB | 0.12 | 0.88 | CC | 111 |
| 12 | BB | BB | BB | BB | 1.00 | 0.00 | NEAC | 88 |
| 13 | BB | BB | BB | BB | 1.00 | 0.00 | NEAC | 102 |
| 14 | AA | AB | AB | BB | 0.00 | 1.00 | CC | 86 |
| 15 | BB | AB | BB | BB | 1.00 | 0.00 | NEAC | 89 |
| 16 | BB | AB | BB | BB | 1.00 | 0.00 | NEAC | 107 |
| 17 | AB | BB | BB | BB | 0.99 | 0.01 | NEAC | 92 |
| 18 | AB | BB | AA | BB | 0.14 | 0.86 | CC | 86 |
| 19 | BB | BB | BB | BB | 1.00 | 0.00 | NEAC | 98 |
| 20 | AB | BB | BB | BB | 0.99 | 0.01 | NEAC | 97 |
| 21 | BB | BB | BB | BB | 1.00 | 0.00 | NEAC | 137 |
| 22 | BB | BB | BB | BB | 1.00 | 0.00 | NEAC | 110 |
| 23 | BB | BB | BB | BB | 1.00 | 0.00 | NEAC | 129 |
| 24 | BB | BB | BB | BB | 1.00 | 0.00 | NEAC | 146 |
| 25 | BB | BB | BB | BB | 1.00 | 0.00 | NEAC | 90 |
| 26 | BB | BB | BB | BB | 1.00 | 0.00 | NEAC | 102 |
| 27 | BB | BB | BB | AB | 1.00 | 0.00 | NEAC | 116 |
| 28 | BB | AB | BB | BB | 1.00 | 0.00 | NEAC | 102 |
| 29 | BB | BB | BB | BB | 1.00 | 0.00 | NEAC | 117 |
| 30 | BB | BB | BB | BB | 1.00 | 0.00 | NEAC | 83 |
| 31 | AB | BB | BB | BB | 0.99 | 0.01 | NEAC | 109 |
| 32 | BB | BB | BB | BB | 1.00 | 0.00 | NEAC | 100 |
| 33 | AB | BB | BB | BB | 0.99 | 0.01 | NEAC | 102 |

**Analysis of size of spawners based on other landing classes (gutted but not beheaded and full fish)**

We analyzed the landings data, to test if the observed trend in size of spawners was dependent on the landing class. Similar to the analysis in the main text, we removed all entries not reported as “cod”, “NEA cod” or “skrei”. Second, we analyzed only entries with a product state of gutted fish but not beheaded (product state code 210, the number of data on full fish was about 30 % compared to the data beheaded and gutted data) or alternatively the full fish weight (product state code 110, the number of data on full fish was about 13 % compared to the data beheaded and gutted data).

Finally, only entries from known spawning grounds in the spawning season were analyzed (see main text for details). The results of these analyses are shown in Figures S2 and S3, and they indicate a similar trend as seen for the gutted and beheaded class.

Figure S2 Weight fraction (corrected for coastal cod in the catch) of very large cod based on gutted but not beheaded fish from the gillnet fishery. The Pearson correlation was r=0.82.

Figure S3 Weight fraction (corrected for coastal cod in the catch) of very large cod based on full fish from the gillnet fishery. The Pearson correlation was r=0.70.

**Analysis of line fishing data**

In the main paper, we focused our analysis on the landings data from gillnets, which were the most abundant gear used (Figure S4). Here, we performed a similar analysis as in the main paper on line fishing data. The results are shown in Figure S5, indicating a similar trend as for the gillnet data (p=0.022, R^2^=0.50, N=10).

Figure S4 show the distribution of gear use in the cod fishery at the spawning grounds.

Figure S5 Similar as Figure 2 in the main text but based on the line fisheries.

**Correction of coastal cod in the landing ticket data**

We used data from the ICES (2017) report to calculate the average size of spawning NEA cod and Coastal cod. Based on these size distributions, we calculated the effect of variable fraction of coastal cod to NEA cod in the catch on the weight fraction of the large size classes. For this calculation, we used the weight at age, the number at age and the fraction of mature at age to calculate the average size frequency distribution of coastal cod and NEA cod for the years 2008-2016. We calculated the fraction of large cod (e.g. larger than 5.0 kg gutted and beheaded in 2008) in a catch with variable ratio of coastal to NEA cod in the population. We used two simple selectivity curves assuming either uniform selectivity for all mature fish or uniform selectivity for all mature fish larger than 2.5 kg (gutted and beheaded). Based on this simple exercise, we conclude that for a 60 % coastal cod in the catch, the weight fraction can be corrected by a multiplicative factor of 1.6 (see Figure S6).

Figure S6 shows the expected weight fractions for uniform selectivity (dotted line) and fishing for the large fish (larger than 2.5 kg gutted and beheaded, solid line) as a function of % NEA cod in the fished population.

**Effects of geographical differences in gillnet mask width**

To address the question to what extent geographic differences in gillnet mask width may affect our results, we investigated data from the coastal reference fleet (Williams 2015). To address this question, we assumed equal size distribution of the spawners across geographical location. Furthermore, we assumed that the mask widths used by the reference fleet (2015-2016) in the different ICES regions (region 07, 06, 00, 05 and 04) were representative of latitudinal trends in mask width in all gill net fisheries for NEA cod. With these assumptions, we quantified the latitudinal trend in the fraction of very large cod caught if gillnet mask width differences were the only factor affecting the variation. Specifically, we calculated the fraction of very large cod expected in the catch if the size distribution was assumed to be equal to the average size distribution from 2008-2016 for all spawning grounds. The results of this analysis are shown as triangles in Figure S7. The results indicate that some, but not all of the trend in size of spawners over latitude can be explained by different gill net mask width.

Figure S7 Solid circles show the latitudinal trend in the weight fraction of very large cod (corrected for bias from coastal cod), as also shown in Figure 2 in the main text. Triangles show the expected values if the mask widths are the same as for the coastal reference fleet and the size distribution is the same for all spawning grounds.

**Testing gear selectivity effects on the genetic data**

To test for potential impact of geographic variations in gear use on the analysis of the individual data, we made an additional analysis based on individual cod caught with gillnet at the spawning grounds in Møre and Lofoten. The cod were caught by a 180 mm mesh gillnet at Møre. In the Møre district, we had available 48 cod of which 20 were identified as NEA cod (see main text). In the Lofoten region, we had a dataset from the 17^th^ of March 2014 obtained with a 188 mm mesh-sized gillnet consisting of 13 individual fish. These fish were not genotyped and hence we could not exclude potential cod of local origin. However, a bias from coastal cod would likely lead to a reduced size in Lofoten compared to Møre, which is a conservative assumption when testing if Lofoten cod is larger than Møre cod. We accounted for the slight gillnet selectivity difference (180 mm in Møre and 188 mm in Lofoten) by adding 4.26 cm to the fish caught in Møre (as expected from the selectivity curves by Huse et al. 2000). We performed a t-test on the log-transformed lengths to test if the NEA cod caught in the Møre region differed in size from the fish caught in the Lofoten region (Figure S). We again found that the cod in Lofoten was significantly larger than the cod in Møre.

Figure S8 shows the boxplot for the two data sets of individual cod from the two spawning grounds obtained with gillnets.

SI References

Barth JMI, Berg PR, Jonsson PR et al. (2017) Genome architecture enables local adaptation of Atlantic cod despite high connectivity. *Molecular Ecology*, **26**, 4452–4466.

Berg PR, Jentoft S, Star B et al. (2015) Adaptation to Low Salinity Promotes Genomic Divergence in Atlantic Cod (*Gadus morhua* L*.*). *Genome Biology and Evolution*, **7**, 1644–1663.

Berg PR, Star B, Pampoulie C et al. (2017) Trans-oceanic genomic divergence of Atlantic cod ecotypes is associated with large inversions. *Heredity*, **119**, 418–428.

Berg PR, Star B, Pampoulie C et al. (2016) Three chromosomal rearrangements promote genomic divergence between migratory and stationary ecotypes of Atlantic cod. *Scientific Reports*, 1–12.

Foll M, Gaggiotti O (2008) A Genome-Scan Method to Identify Selected Loci Appropriate for Both Dominant and Codominant Markers: A Bayesian Perspective. *Genetics*, **180**, 977–993.

Huse I, Løkkeborg S, Soldal AV (2000) Relative selectivity in trawl, longline and gillnet fisheries for cod and haddock. *ICES Journal of Marine Science*.

ICES (2017) Report of the Arctic Fisheries Working Group (AFWG). ICES CM 2017/ACOM:06, 1–493.

Kirubakaran TG, Grove H, Kent MP et al. (2016) Two adjacent inversions maintain genomic differentiation between migratory and stationary ecotypes of Atlantic cod. *Molecular Ecology*, **25**, 2130–2143.

Sodeland M, Jorde PE, Lien S et al. (2016) “Islands of Divergence” in the Atlantic Cod Genome Represent Polymorphic Chromosomal Rearrangements. *Genome Biology and Evolution*, **8**, 1012–1022.

Williams T. Årsrapport 2015 Havforskningsinstituttets referanseflåte. Institute of Marine Research; 2016 Jul pp. 1–19 (In Norwegian).
